# Histological characteristics, cell wall hydrolytic enzyme activity, and transcriptome analysis with seed shattering of *Stylosanthes* accessions

**DOI:** 10.3389/fpls.2022.1018404

**Published:** 2022-10-17

**Authors:** Xinyong Li, Jingwen Zhang, Jingxue Zhang, Wei Sheng, Rui Huang, Rongshu Dong, Xipeng Ding, Pandao Liu, Guodao Liu

**Affiliations:** ^1^ Institute of Tropical Crop Genetic Resources, Chinese Academy of Tropical Agricultural Sciences, Danzhou, China; ^2^ Ministry of Education Key Laboratory for Ecology of Tropical Islands, College of Life Science, Hainan Normal University, Haikou, China

**Keywords:** *Stylosanthes*, seed shattering, cell wall degrading, histological characteristics, transcriptome analysis

## Abstract

*Stylosanthes* spp. (stylo) are annual or perennial legume forages that are widely grown as forage and cover crops in tropical and subtropical regions. However, the seed yield of stylo is very low due to serious seed shattering. In the present study, we found that, although seed shattering was common among the stylo accessions, the shattering rates were genetically different. Therefore, we first synthesized the morphological, histological characteristic, physiochemical, and transcriptome analyses to determine the seed shattering mechanism in stylo. In general, the stylo germplasm with shorter lobules and thicker stems had a lower seed shattering rate and a higher seed weight. The seed and seed stalk joint is the abscission zone in stylo. Multiplex histology and hydrolytic enzyme activity analysis showed that the tearing of the abscission zone occurs due to the intense enzymatic degradation of polygalacturonase and cellulase in the seed shattering-susceptible accession TF0275. cDNA libraries from the abscission zone tissues of TF0041 and TF0275 at 14, 21, and 28 days after flowering were constructed and sequenced. A total of 47,606 unigenes were annotated and 18,606 differentially expressed genes (DEGs) were detected, including 9,140 upregulated and 9,446 downregulated unigenes. Furthermore, the 26 candidate DEGs involved in lignin biosynthesis, cellulase synthesis, and plant hormone signal transduction were found at all three developmental stages. This study provides valuable insights for future mechanistic studies of seed shattering in stylo.

## Introduction

Seed shattering (SS) is essential for the dispersal and persistence of seeds in many wild plants (Zhao et al., 2015). However, SS had been undesirable since the beginning of the domestication of crops, with consistent efforts having been made to minimize it due to its adverse effect on seed yield (Funatsuki et al., 2006; Lin et al., 2007). However, studies of the mechanisms of SS are scarce for most wild and forage varieties because, for breeders, the biomass yield is usually more important than the seed yield, although SS is a highly diverse trait in many forage varieties (Maity et al., 2021). Improved seed retention ability is necessary for high SS cultivated forage grasses.

SS is a highly complex event involving genetic and physiological mechanisms (Zhao et al., 2019). In many crops, cleavage of the abscission layers formed at seed bases leads to SS (Fuller et al., 2009). Moreover, abscission is related to the degradation of abscission layer cells by cellulase (CE) and polygalacturonase (PG) (Henderson et al., 2001; Roberts et al., 2002). The CE concentration in the abscission zone plays a major role in determining organ abscission (Roberts et al., 2002). PG can hydrolyze pectin in the abscission zones, which leads to cell dissolution during plant development (Daher and Braybrook, 2015). Swelling and degradation of the abscission zone cells are accomplished by certain genes. In rice, some major shattering genes have been identified, including *sh-2*, *SH4*, *SHAT1*, and *qSH1* (Oba et al., 1995; Li et al., 2006; Konishi et al., 2006; Zhou et al., 2012). The *Q* gene is the major gene that regulates seed dispersal in wheat (Simons et al., 2006), and a shattering quantitative trait locus (QTL) *Sh1*-orthologous gene was identified in maize (Lin et al., 2012).

*Stylosanthes* spp. (stylo) are annual or perennial legume forages widely grown as forage and cover crops in tropical and subtropical regions (Schultze-Kraft et al., 2018; Guo et al., 2019). When harvest is delayed, SS might result in 60% seed losses of stylo in the field. Therefore, improving the seed retention ability is very important for stylo seed production. However, studies of the SS mechanisms in stylo are limited, especially in ecology, physiology, and genetics. This is the first study undertaking morphological and histological characteristic analyses, hydrolytic enzyme activity determination, and transcriptome analysis to examine the SS mechanism of stylo accessions. The aims of this study were to i) determine the SS rate of stylo; ii) identify the location and timing of the abscission zone; iii) identify the cell wall hydrolytic enzymes active in SS; and iv) identify candidate SS genes.

## Materials and methods

### Plant materials

The 68 stylo accessions used in this study were sown at the Institute of Tropical Crop Genetic Resources, Chinese Academy of Tropical Agriculture Sciences, Hainan, China (19°30′ N, 109°30′ E) (**Table 1**). The mean annual precipitation is 2,229 mm.

**Table 1:** Sixty-eight stylo accessions used in this study.

Code	Accession	Latin name	Code	Accession	Latin name
1	TF0027	*Stylosanthes seabrana*	35	TF0051	*Stylosanthes hamata*
2	TF0041	*Stylosanthes grandifolia*	36	TF0286	*Stylosanthes guianensis*
3	TF0074	*Stylosanthes humilis*	37	TF0321	*S. guianensis*
4	TF0035	*Stylosanthes gracilis*	38	TF0287	*S. guianensis*
5	TF0126	*S. seabrana*	39	TF0225	*S. guianensis*
6	TF0145	*Stylosanthes viscosa*	40	TF0256	*S. guianensis*
7	TF0144	*S. viscosa*	41	TF0175	*S. guianensis*
8	TF0058	*S. hamata*	42	TF0306	*S. guianensis*
9	TF0276	*S. guianensis*	43	TF0305	*S. guianensis*
10	TF0285	*Stylosanthes scabra*	44	TF0177	*S. guianensis*
11	TF0054	*S. hamata*	45	TF0208	*S. guianensis*
12	TF0042	*S. grandifolia*	46	TF0227	*S. guianensis*
13	TF0188	*S. guianensis*	47	TF0224	*S. guianensis*
14	TF0307	*S. guianensis*	48	TF0243	*S. guianensis*
15	TF0050	*S. hamata*	49	TF0205	*S. guianensis*
16	TF0172	*S. guianensis*	50	TF0274	*S. guianensis*
17	TF0275	*S. guianensis*	51	TF0223	*S. guianensis*
18	TF0271	*S. guianensis*	52	TF0057	*S. hamata*
19	TF0322	*S. guianensis*	53	TF0222	*S. guianensis*
20	TF0261	*S. guianensis*	54	TF0186	*S. guianensis*
21	TF0260	*S. guianensis*	55	TF0290	*S. guianensis*
22	TF0272	*S. guianensis*	56	TF0110	*S. scabra*
23	TF0244	*S. guianensis*	57	TF0171	*S. guianensis*
24	TF0259	*S. guianensis*	58	TF0206	*S. guianensis*
25	TF0273	*S. guianensis*	59	TF0258	*S. guianensis*
26	TF0207	*S. guianensis*	60	TF0228	*S. guianensis*
27	TF0019	*Stylosanthes capitata*	61	TF0211	*S. guianensis*
28	TF0191	*S. guianensis*	62	TF0289	*S. guianensis*
29	TF0012	*S. capitata*	63	TF0163	*S. subsericea*
30	TF0187	*S. guianensis*	64	TF0209	*S. guianensis*
31	TF0156	*Stylosanthes mexicana*	65	TF0192	*S. guianensis*
32	TF0257	*S. guianensis*	66	TF0115	*S. scabra*
33	TF0242	*S. guianensis*	67	TF0210	*S. guianensis*
34	TF0226	*S. guianensis*	68	TF0255	*S. guianensis*

### Seed shattering and seed shattering-related trait measurements

The SS rate of the 68 accessions was measured using the bagging method. Twenty inflorescences of each plant were bagged after flowering, and the seeds in the bag were examined at 28 days after flowering (DAF) to calculate the SS rates in two consecutive years (2020–2021). Six other SS-related traits, including lobular length (LL), lobular width (LW), plant height (PH), stem diameter (SD), number of main branches (NMB), and 1,000-seed weight (TKW), were measured using the methods described by Zhang et al. (2022). Data were subjected to analysis with the coefficient of variation (CV) and variance using SPSS 19.0 (SPSS Inc., Chicago, IL, USA) software. Significance was set at *p* < 0.05. Clustering was conducted using the methods described by Deng et al. (2014).

### Multiplex histology analysis of the abscission zone

A total of two accessions were used in this study, TF0041 and TF0275, which were selected based on the SS results in the 68 stylo accessions. Histological analysis of the seed abscission zone using frozen sections was carried out at each of the five developmental stages: 7, 14, 21, 28, and 35 DAF. During the sampling period, the seed stalks from the two accessions were saved in FAA (formaldehyde alcohol acetic acid) fixation solution. Subsequently, an OCT embedding agent was used to embed the seed stalk and a cryostat used to dissect 10-μm sections. The sections were stained with 0.5% toluidine blue for 20 min. An OLYMPUS BX51 microscope was used to analyze the images.

Seeds and seed stalks were collected from the two accessions and from five developmental stages. The samples were then sputter-coated with gold and palladium for 60 s. After these treatments, the junctions of the seed and seed stalk were observed with scanning electron microscopy.

### Hydrolytic enzyme analysis of the abscission zone

The CE and PG enzyme activities in the abscission layer of the two accessions (TF0041 and TF0275) were assayed using a plant CE and PG ELISA kit at the same five developmental stages.

### RNA isolation, cDNA library construction, and illumina sequencing

Based on the results of the histological and hydrolytic enzyme analyses, 40 mg of abscission zone tissues (1 mm region of the seed stalk and 2 mm of the seed) from the two accessions (TF0041 and TF0275) was collected at 14, 21, and 28 DAF for testing in three biological replications. These samples were immediately frozen in liquid nitrogen and stored at −80°C until RNA isolation. A Plant Total RNA Extraction Kit was used to isolate the total RNA from each sample. An Agilent 2100 Bioanalyzer was employed to measure the RNA concentration and quality. Oligo(dT) beads were used for Poly(A) messenger RNA (mRNA) enrichment. Complementary DNA (cDNA) libraries were constructed using the Illumina TruSeq RNA Library Prep Kit and were sequenced using an Illumina NovaSeq 6000 platform from Biomarker Co. Ltd. (Beijing, China) with a read length of 150 bp.

### Functional annotation

The raw sequencing reads were filtered to obtain clean reads. Furthermore, the Trinity program was used to assemble clean reads (Grabherr et al., 2011). For annotation, all unigene sequences were analyzed according to Jia et al. (2020).

### Analysis of differentially expressed genes

The assembled transcripts were aligned against the unigene library using Bowtie2, and expression was calculated and represented as reads of fragments per kilobase of transcript sequence per million base pairs sequenced (FPKM) using RNA sequencing (RNA-seq) data with expectation maximization (RSEM) (Li and Dewey, 2011). DESeq2 was utilized to calculate the differences in the expression abundance between three developmental stages (Anders and Huber, 2010), for which thresholds of log2 (fold change) >1 and *p* ≤ 0.05 were considered for the classification of differentially expressed genes (DEGs) (Liu et al., 2016).

## Results

### Seed shattering degree of the 68 stylo accessions

**Figure 1:**
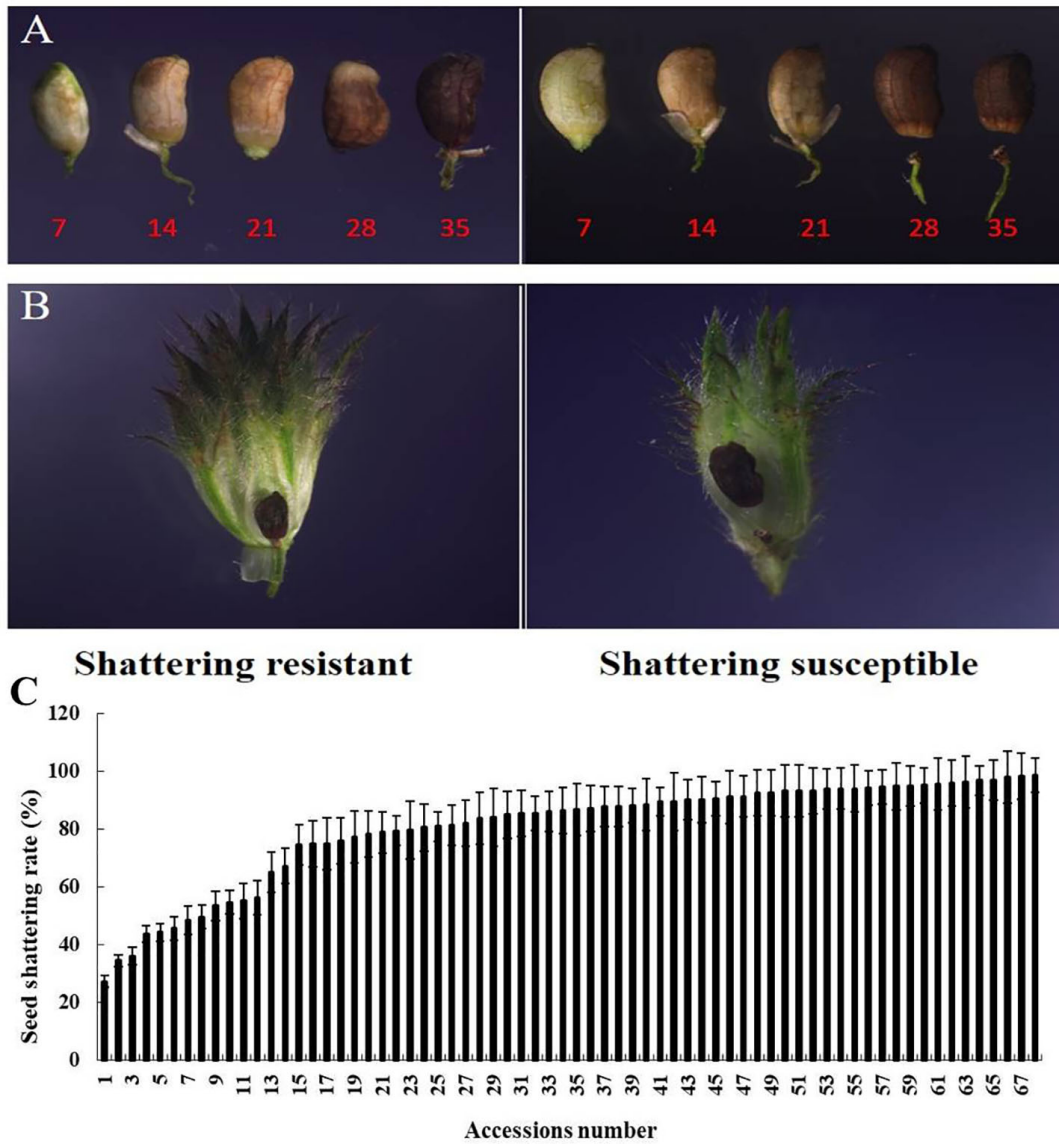
Different seed shattering (SS) habits of the stylo accessions. **(A)** Time course changes in the SS position of the SS-resistant and SS-susceptible accessions at 7, 14, 21, 28, and 35 days after flowering (DAF). **(B)** SS position of the SS-resistant and SS-susceptible accessions at 28 DAF in the inflorescence. **(C)** Average SS rate of the 68 stylo accessions. *Bars* indicate the mean ± SD.

In this study, the SS rates of the 68 stylo accessions varied from 27% to 99%, with an average of 76% (**Figure 1C**). Seven accessions had relatively low SS rates, with the other 61 accessions having SS rates higher than 50%, which indicated that the phenomenon of SS was common in the stylo accessions. We found that seeds separated from seed stalks at 28 DAF in the field (**Figures 1A, B**).

### Morphological variation in the 68 stylo accessions

**Figure 2:**
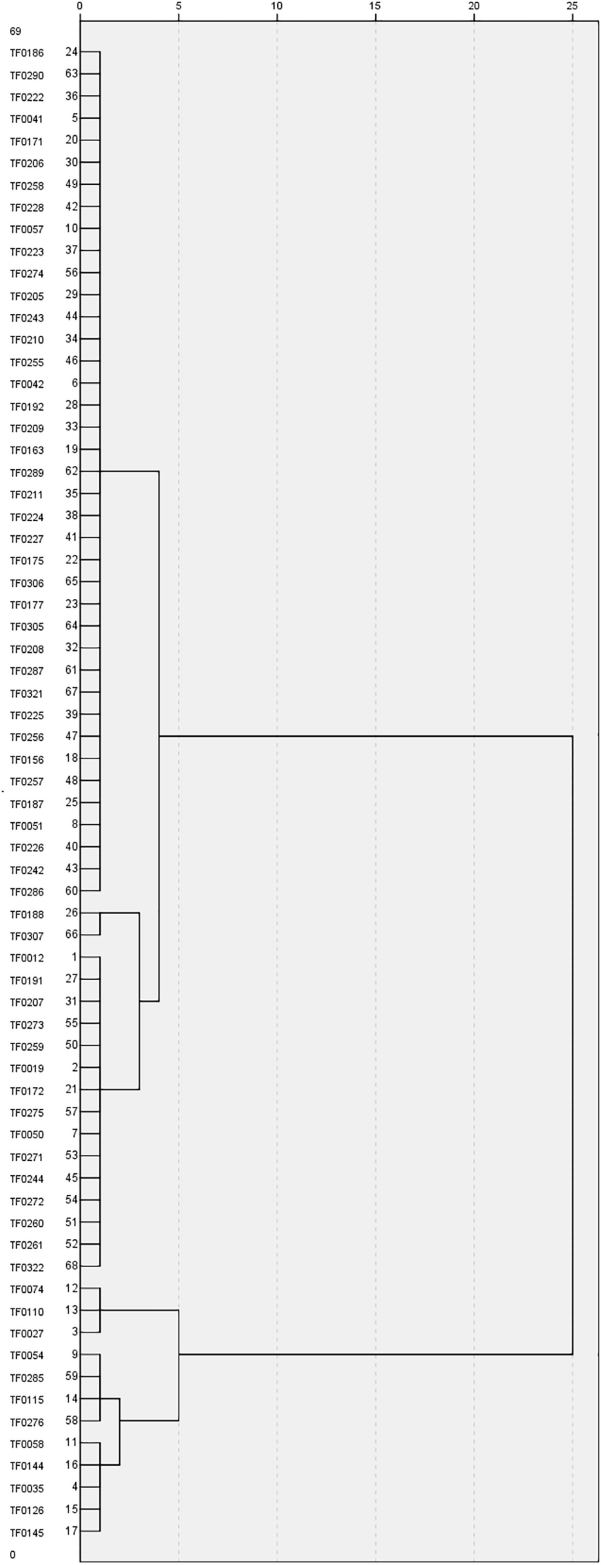
Clustering analysis of the 68 stylo accessions based on morphological traits. Accession names are shown on the *left side* of the dendrogram.

Morphological variations in the 68 stylo accessions were studied using seven SS-related traits: SS, LL, LW, PH, SD, NMB, and TKW. Three of these seven traits—PH, SD, and NMB—had CV values greater than 40%. The greatest morphological variation was found for NMB (67.2%), followed by SD (48.6%), PH (40.1%), TKW (28.6%), LL (27.7%), LW (24.2%), and SS (22.3%) (**Table 2**). SS showed a significant positive correlation with LL and a significant negative correlation with SD and TKW (**Table 3**). Based on the SS-related traits, clustering analysis classified the 68 stylo accessions into two different groups (**Figure 2**). The first group included 56 accessions with SS rates higher than 65%, while the second group comprised 12 accessions with SS rates lower than 60%. The morphological variability was concordant with the dendrogram.

**Table 2:** Morphological variations in the seven seed shattering-related traits among the 68 stylo accessions.

Traits	Min	Max	Mean	SD	CV (%)
LL (mm)	12.5	46.2	28.2	7.8	27.7
LW (mm)	2.6	11.6	6.2	1.5	24.2
PH (cm)	20.7	139.9	71.4	28.6	40.1
SD (mm)	1.5	10.0	3.5	1.7	48.6
NMB	9.0	161.0	50.0	33.6	67.2
TKW (g)	0.8	2.5	1.4	0.4	28.6
SS (%)	27.3	98.8	80.3	17.9	22.3

LL, lobular length; LW, lobular width; PH, plant height; SD, stem diameter; NMB, number of main branch; TKW, 1,000-seed weight; SS, seed shattering; SD, standard deviation; CV, coefficient of variation

**Table 3:** Correlation analysis between seed shattering and other seed shattering-related traits.

Traits	LL	LW	PH	SD	NMB	SS	TKW
LL	1.000						
LW	0.516**	1.000					
PH	0.519**	0.422**	1.000				
SD	−0.337**	−0.042	0.149	1.000			
NMB	−0.096	0.199	−0.189	0.035	1.000		
SS	0.401**	0.063	−0.002	−0.654**	−0.002	1.000	
TKW	−0.144	0.044	0.011	0.255*	0.173	−0.260*	1.000

LL, lobular length; LW, lobular width; PH, plant height; SD, stem diameter; NMB, number of main branch; TKW, 1,000-seed weight; SS, seed shattering.

* means significant correlation at *p*<0.05 level; ** means significant correlation at *p*<0.01 level.

### Histological analysis

**Figure 3:**
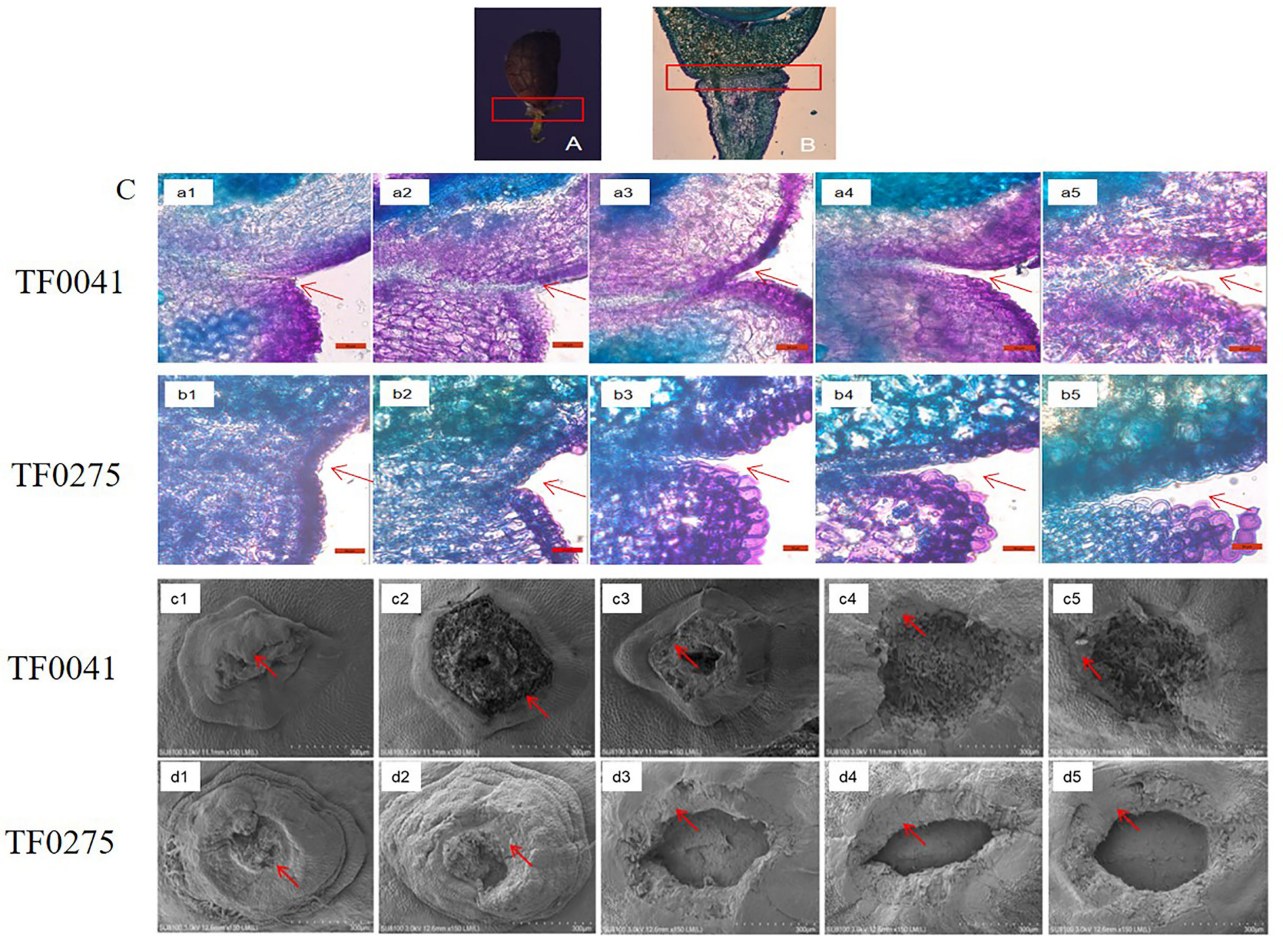
Histological analysis of the abscission zone. **(A, B)** Seed stalk histological structure and abscission layer position in the seed stalk. **(C)** *a1* and *b1*, *a2* and *b2*, *a3* and *b3*, *a4* and *b4*, and *a5* and *b5* show the longitudinal sections across the abscission zones of TF0041 and TF0275 at 7, 14, 21, 28, and 35 days after flowering (DAF), respectively. *c1* and *d1*, *c2* and *d2*, *c3* and *d3*, *c4* and *d4*, and *c5* and *d5* display the scanning electron microscopy photos of the seed stalk junctions after detachment of the seeds in TF0041 and TF0275 at 7, 14, 21, 28, and 35 DAF, respectively. The *red arrow* indicates the location of the abscission layer. The *red frame* in **(A)** and **(B)** denotes the abscission zone. *Bar*, 50 μm in *a1*–*a5* and *b1*–*b5* and 300 μm in *c1*–*c5* and *d1*–*d5*.

Based on the SS results of the 68 stylo accessions, we selected two accessions for further study: TF0041 (an SS-resistant accession) and TF0275 (an SS-susceptible accession). Histological investigation of the longitudinal sections indicated that abscission layers occurred on both sides of the seed stalk (**Figures 3A, B**). The parenchyma cells were larger than the abscission layer cells in the seed stalk and had a thin cell wall (**Figure 3C**, a1 and b1). The abscission layer was not degraded in the two accessions by day 14 after flowering (**Figure 3C**, a2 and b2). Degradation of adjacent cell walls in the abscission layer was found in TF0275 at 21 DAF (**Figure 3C**, b3), and the cleavage abscission layer occurred at 28 DAF (**Figure 3C**, b4). The endopleura contours became blurred. In comparison, serious swelling and dissolution of the abscission layer were found in TF0041 at 35 DAF (**Figure 3C**, a5), which were not observed at 21 or at 28 DAF (**Figure 3C**, a3 and a4, respectively). Early disruption of the abscission cells resulting in the tearing of the abscission layer increased the release of seeds in the high SS accession TF0275. The staining results revealed more lignin in the abscission zone of TF0275 than that in TF0041 during the seed developmental stages (**Figure 3C**). The scanning electron microscopy results showed that the rupture surface center vascular bundle was convex within 14 DAF, but as the seed developed and matured, the rupture surface center gradually became concave (**Figure 3C**). The seed stalk in TF0275 had a smooth fracture surface and a smaller intercellular space at 21, 28, and 35 DAF (**Figure 3C**, d3, d4, and d5), while a dimpled appearance was found in TF0041; the cellular contour was visible (**Figure 3C**, c3, c4, and c5).

### Analysis of hydrolytic enzymes

**Figure 4:**
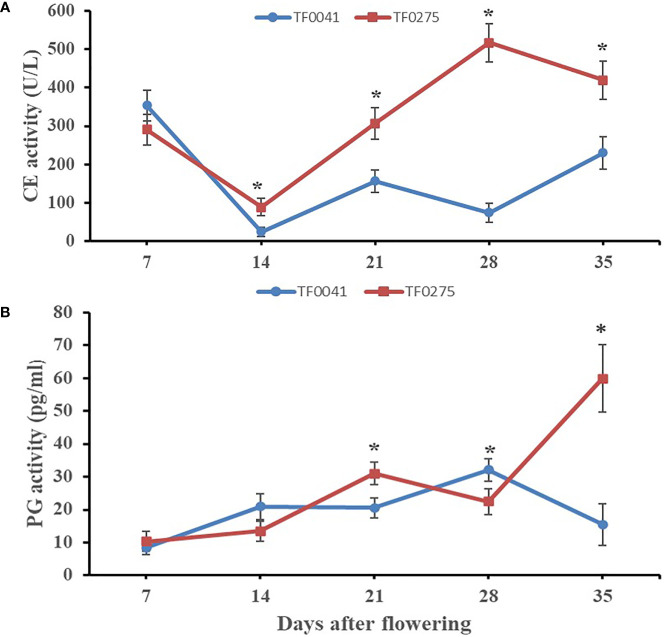
Specific activity of two cell wall-degrading enzymes, cellulase (CE) **(A)** and polygalacturonase (PG) (**B**), in the abscission zone. *Bars* indicate the mean ± SD. *Asterisk* indicates a significant difference in the enzyme activity between TF0041 and TF0275 at the *p* < 0.05 level.

To investigate the association between hydrolytic enzymes and SS, we assayed the activities of CE and PG (**Figures 4A, B**) in the abscission zones of TF0041 and TF0275. The CE activity of the two accessions showed different trends at different developmental stages. Generally, the average activity of CE was lower in the SS-resistant accession TF0041 (168.00 U/L) than that in the SS-susceptible accession TF0275 (324.69 U/L). The largest differences in the CE activity between the two accessions were observed at 28 DAF. The CE activity increased rapidly at 14 DAF and reached 516.92 U/L at 28 DAF in TF0275, while it was only 74.12 U/L at 28 DAF in TF0041. The TF0041 accession exhibited a lower average PG activity (19.49 pg/ml) than TF0275 (27.41 pg/ml). At 35 DAF, the PG activity of TF0275 was 59.87 pg/ml, which was nearly four times that of TF0041 (15.53 pg/ml). Hydrolytic enzyme analysis indicated significantly different cell wall-degrading enzyme activities between TF0275 and TF0041 in the abscission zone at 21, 28, and 35 DAF.

### Transcriptome sequencing analysis

To explore the genetic mechanism and candidate genes modulating SS in stylo, we used RNA-seq analysis to profile the gene expression in three developmental stages. The raw data files have been submitted to NCBI under BioProject accession number PRJNA860716. After processing, the number of clean reads was between 7.2 and 10.8 million (**Table 4**). We identified 220,873 unigenes and annotated 47,606 unigenes in all databases (**Table 5**). To further construct a detailed regulatory network for SS in different developmental stages, we detected more than 20,000 DEGs at 14, 21, and 28 DAF. Comparison of the DEGs revealed 8,372 (3,649 upregulated and 4,723 downregulated) DEGs in TF0041-14 *vs*. TF0275-14, 5,980 (3,663 upregulated and 2,317 downregulated) DEGs in TF0041-21 *vs*. TF0275-21, and 4,254 (1,828 upregulated and 2,426 downregulated) DEGs in TF0041-28 *vs*. TF0275-28 (**Table 6**).

**Table 4:** Summary of the sequence data analysis.

Sample	Total clean reads (bp)	Total clean nucleotides (Gb)	GC%	≥*Q*_30_ (%)
TF0275-14-1	9,301,789	2.79	42.64	93.12
TF0275-14-2	9,771,410	2.93	43.41	93.86
TF0275-14-3	7,238,508	2.17	43.47	93.87
TF0275-21-1	8,926,721	2.68	42.74	93.6
TF0275-21-2	8,008,786	2.4	41.36	93.89
TF0275-21-3	9,612,892	2.88	42.87	93.99
TF0275-28-1	7,820,780	2.35	42.11	93.94
TF0275-28-2	10,469,427	3.14	42.54	93.8
TF0275-28-3	10,249,689	3.07	42.83	94
TF0041-14-1	8,526,285	2.56	43.93	93.19
TF0041-14-2	9,744,237	2.92	44	93.91
TF0041-14-3	10,807,099	3.24	43.89	93.83
TF0041-21-1	9,498,871	2.85	43.49	93.8
TF0041-21-2	10,038,798	3.01	43.83	93.1
TF0041-21-3	10,724,834	3.22	43.45	93.65
TF0041-28-1	9,167,897	2.75	43.19	93.89
TF0041-28-2	7,569,946	2.27	42.2	93.59
TF0041-28-3	7,922,941	2.38	43.4	93.84

**Table 5:** BLAST analysis of the non-redundant unigenes against public databases.

Annotated database	No. of unigenes	300 ≤ length < 1,000	Length ≥ 1,000
Nr annotation	198,069	37,412	160,458
Nt annotation	208,414	46,966	160,226
KO annotation	102,449	16,647	85,787
SwissProt annotation	172,514	27,817	144,664
Pfam annotation	172,639	31,466	139,980
GO annotation	172,619	31,465	139,961
KOG annotation	63,944	9,904	54,037
Annotated in all database	47,606	7,259	40,345

Nr, NCBI non-redundant protein sequences; Nt, NCBI non-redundant nucleotide sequences; KO, Kyoto Encyclopedia of Genes and Genomes Ortholog; PFAM, Protein Family database; GO, Gene Ontology; KOG, Eukaryotic Ortholog Groups.

**Table 6:** Statistical table of the differently expressed transcripts (DEGs), with annotation.

Type	TF0041-14 *vs*. TF0275-14	TF0041-21 *vs*. TF0275-21	TF0041-28 *vs*. TF0275-28
*N*	8,372	5,980	4,254
Up	3,649	3,663	1,828
Down	4,723	2,317	2,426
Nr	7,786	5,672	3,918
Nt	7,889	5,757	3,944
KO	3,456	2,320	1,744
SwissProt	6,675	4,863	3,359
Pfam	6,657	4,864	3,335
GO	1,738	1,116	919
KOG	2,224	1,406	1,240
Annotated in all database	1,716	998	877

Up, upregulated; Down, downregulated; Nt, NCBI non-redundant nucleotide sequences; KO, Kyoto Encyclopedia of Genes and Genomes Ortholog; PFAM, Protein Family database; GO, Gene Ontology; KOG, Eukaryotic Ortholog Groups.

### Functional annotation and classification

**Figure 5:**
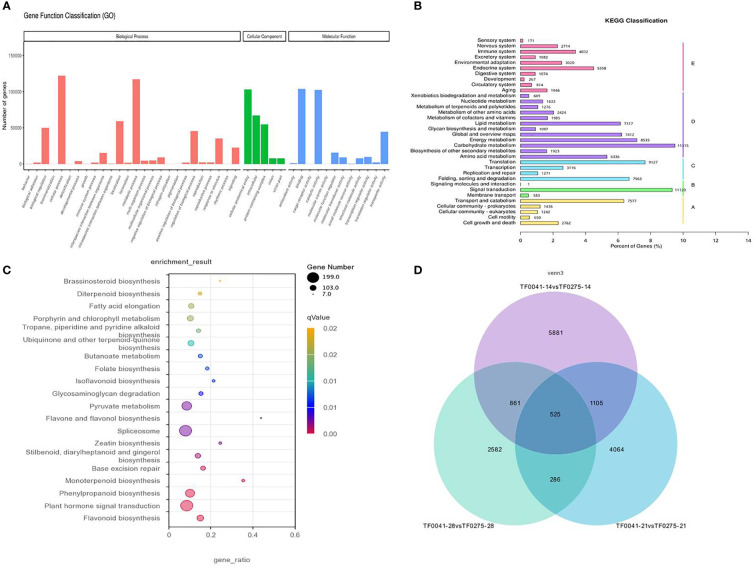
Classification of the assembled transcripts and the differentially expressed genes (DEGs). **(A)** Gene Ontology (GO) classification of the assembled transcripts. **(B)** Kyoto Encyclopedia of Genes and Genomes (KEGG) classification of the assembled transcripts. **(C)** KEGG classification of the DEGs. **(D)** Venn diagram representing the number of DEGs consistently present in the three groups.

To understand the biological processes involved in the SS of stylo, we performed Gene Ontology (GO) analysis. The 172,619 unigenes were annotated to three main GO categories (molecular function, biological process, and cellular component) (**Figure 5A**). In the molecular function category, “catalytic activity” and “transporter activity” were the dominant terms. In the biological process category, “cellular process” and “metabolic process” were the dominant groups. In the cellular component category, “intracellular,” “DNA-directed RNA polymerase complex,” and “transferase complex” were the dominant groups.

The annotated genes were aligned against the Kyoto Encyclopedia of Genes and Genomes (KEGG) database to explore pathways. As a result, a total of 102,449 annotated genes were assigned to 34 KEGG pathways. Notably, the largest category classified by KEGG was that of metabolic pathways, which were primarily abundant, with 51,562 annotated genes. These were further classified into 12 subcategories, and the significantly enriched subcategories were carbohydrate metabolism (11,315), energy metabolism (8,535), global and overview maps (7,412), and lipid metabolism (7,317) (**Figure 5B**).

### Candidate genes

To select candidate genes related to SS, all the DEGs from three differentially expressed transcript sets were also subjected to KEGG pathway analysis. As a result, 868 DEGs could be annotated and assigned to a KEGG pathway, with the most representative pathway found including “flavonoid biosynthesis (ko00941),” “plant hormone signal transduction (Ko4075),” “phenylpropanoid biosynthesis (Ko00940),” and “monoterpenoid biosynthesis (ko00902),” among others (**Figure 5C**). The results of the histological and hydrolytic enzyme analyses showed that cell wall degradation resulting in the tearing of the abscission layer increased the seed release in the SS-susceptible accession TF0275, which also had significantly higher CE and PG activities at 21 and 28 DAF compared to the SS-resistant accession TF0041. Therefore, we mainly focused on the KEGG pathways associated with cell wall degradation in this study, which included “plant hormone signal transduction (Ko4075),” “phenylpropanoid biosynthesis (Ko00940),” and “peroxisome (Ko04146).” In the “plant hormone signal transduction” pathway, 139 unigenes were annotated. In the “phenylpropanoid biosynthesis” pathway, 87 unigenes were annotated, which are involved in lignin biosynthesis (**Supplementary Table S1**).

To narrow down the list of candidate genes related to SS, we targeted the 525 DEGs that were consistently present in the three groups of DEGs (**Figure 5D**) and performed KEGG pathway analysis. In particular, one unigene involved in abscisic acid (m54237_190703_092441_34734586_ccs) was downregulated, while four and two unigenes respectively involved in auxin (m54237_190703_092441_33096305_ccs, m54237_190703_092441_23134998_ccs, m54237_190703_092441_23527840_ccs, and m54237_190703_092441_70124061_ccs) and ethylene (m54237_190703_092441_65471267_ccs and m54237_190703_092441_13239270_ccs) were upregulated in the SS-susceptible accession TF0275 compared to the SS-resistant accession TF0041. Sixteen DEGs involved in cell wall hydrolase were predicted, of which two unigenes involved in cellulose synthase (m54237_190703_092441_26738903_ccs and m54237_190703_092441_37290330_ccs) were downregulated and eight unigenes involved in pectinesterase enzyme (m54237_190703_092441_57934234_ccs, m54237_190703_092441_29557596_ccs, m54237_190703_092441_63111712_ccs, m54237_190703_092441_4522764_ccs, m54237_190703_092441_9307057_ccs, m54237_190703_092441_49611125_ccs, m54237_190703_092441_20054802_ccs, and m54237_190703_092441_43844162_ccs) were upregulated in TF0275. Three DEGs involved in phenylpropanoid biosynthesis were predicted, of which one unigene involved in peroxidase (m54237_190703_092441_58721034_ccs) was downregulated and two unigenes involved in peroxidase β-glucosidase (m54237_190703_092441_56099453_ccs) and 4-coumarate-CoA ligase (m54237_190703_092441_41288657_ccs) were upregulated in TF0275 (**Table 7**). These 26 candidate DEGs involved in lignin biosynthesis, CE synthesis, and plant hormone signal transduction were found at all three developmental stages. In addition to these candidate SS-related genes, other DEGs might influence SS interactions in the stylo.

**Table 7:** Twenty-six candidate differentially expressed genes (DEGs) enriched in plant hormone signal transduction, cell wall hydrolase, and phenylpropanoid biosynthesis pathway.

KEGG pathway	KO name	KO description	KO i.d.	Total	Up	Down
Plant hormone signal transduction						
Abscisic acid	CTR1	Serine/threonine protein kinase CTR1	K14510	1	0	1
Auxin	SAUR	SAUR family protein	K14488	4	4	0
Ethylene	EIN3	Ethylene-insensitive protein 3	K14514	1	1	0
	EBF1_2	EIN3-binding F-box protein	K14515	1	1	0
Cell wall hydrolase						
Cellulose synthase	CESA	Cellulose synthase A	K10999	6	3	3
	CSLD	Cellulose synthase-like protein	K20924	2	1	1
Pectinesterases enzyme	E3.1.1.11	Pectinesterase	K01051	8	8	0
Phenylpropanoid biosynthesis						
	POX	Peroxidase	K00430	1	0	1
	BGLU	Beta-glucosidase	K01188	1	1	0
	4CL	4-Coumarate–CoA ligase	K01904	1	1	0

KEGG, Kyoto Encyclopedia of Genes and Genomes; KO, KEGG Ortholog; Up, upregulated; Down, downregulated; NO.All, total.

## Discussion

### Correlation of seed shattering-related traits

SS is an important trait in pasture grasses (Dong and Wang, 2015). In this study, it was found that more than 85% of the accessions had SS rates higher than 50%, which indicated that the phenomenon of SS was common in the stylo accessions. We also discovered a significant correlation between SS and three other SS-related traits—LL, SD, and TKW—which indicated that the stylo germplasm with shorter lobules and thicker stems had a lower SS rate and a higher seed weight. Similar to the results of McWilliam (1980), many factors influence SS, such as seed size, weight, and the number of branches. Therefore, the collection of wider germplasms and recurrent selection with those agronomic traits in practice, as well as choosing germplasms with shorter lobules and thicker stems, will be important to improve seed retention in this species. This study found that, when seeds usually fall from the seed stalk, the seed stalk was still on the inflorescence in the field. Therefore, the seed and the seed stalk joint is the abscission zone in stylo.

### Histological and hydrolytic enzyme activity differences in the abscission zone

Previous studies have shown that SS can usually be divided into two stages: the first being the formation of the abscission layer and the second the degradation of the abscission layers (Li et al., 2006; Akasaka et al., 2011). Additionally, the degradation of the abscission layers occurs due to enzymatic deterioration. Cellulose, as a main fiber, provides strength and structural integrity to plant cells, which can be hydrolyzed by CE to directly affect shattering (Roberts et al., 2002; Muriira et al., 2015). Pectin is the main component of plant cell walls and intercellular layers, which have important consequences for cell adhesion, and can be hydrolyzed by PG (Henderson et al., 2001; Liljegren, 2012). Subsequently, dissolving pectin between adjacent cell walls in the abscission zone allows protoplasts to collapse (Christiansen et al., 2002).

To explore the development of the abscission zone in stylo, we synthesized the physiochemical and multiplex histology analyses in the two accessions (TF0041 and TF0275). Multiplex histology analysis of the abscission zone showed that higher levels of dissolution of the abscission layer cells led to the tearing of the abscission layer and that the surface of the seed stalk appeared smooth in TF0275 after 14 DAF. This may be due to the higher PG and CE activities in the development of the abscission zone of TF0275, similar to previous reports (Henderson et al., 2001; Tada et al., 2015). These results indicated that the activities of PG and CE increased significantly during seed development in the SS-sensitive accessions, which was consistent with the degree of abscission layer cell degradation. This may be caused by the interaction between PG and CE, in which PG hydrolyzes the cell wall pectin, leading to a decreased pH, which could elevate CE activity to reduce the structural integrity of plant cells (Willats et al., 2001).

### Candidate genes involved in seed shattering

SS is regulated by complex polygenic traits and their interactions (Dong and Wang, 2015). Pectin and cellulose are the main components of the plant cell wall, and their hydrolysis and metabolism have a significant effect on cell wall degradation (Dong et al., 2017). Many plant CE genes alter the structure and composition of the cell wall during tissue development (Libertini et al., 2004). In the pectin metabolism pathway, pectin is de-esterified by pectinesterase hydrolysis (Zhang et al., 2015), and the pectinases ADPG1 and ADPG2 are essential for silique dehiscence in *Arabidopsis thaliana* (Ogawa et al., 2009). The KEGG pathway analysis for the DEGs in this study showed that eight unigenes were involved in CE activity and another eight were involved in pectate lyase. Most of these unigenes were upregulated in both accessions at 28 DAF. Interestingly, compared to the SS-resistant accession, two unigenes (m54237_190703_092441_26738903_ccs and m54237_190703_092441_37290330_ccs) were determined to be involved in cellulose synthesis, the expression levels of which were significantly lower in the SS-susceptible accession. This result showed that the catabolism of cellulose splitting increased and anabolism decreased, which was consistent with physiological data (**Figure 4**), indicating aggravation of the breakdown of the abscission layer cells in the SS-susceptible accession. Our results are similar to those of Dong et al. (2017).

In addition to cell wall hydrolytic enzymes, plant hormones also play a major role in regulating the development processes of the abscission zone as signal molecules. For example, auxin, ethylene, and abscisic acid play direct or indirect roles in SS (Sargent et al., 1984; Taylor and Whitelaw, 2001). Additionally, the production and activity of many plant hormones and specific enzymes are triggered by certain genes. A number of abscisic acid (ABA)-responsive genes (e.g., *PP2C*, *PYR/PYL*, *ABF*, *SnRK2*, and *NCED*) regulate the signal transduction of ABA (Fujii et al., 2009; Lang et al., 2021). The ethylene-insensitive mutant of *Arabidopsis etr1* exhibited a delay in the shedding of floral parts (Schaller and Bleecker, 1995). In the present study, six unigenes related to plant hormone signal transduction were upregulated in the SS-susceptible accession, indicating that the interaction of these genes may have contributed to SS.

Lignin is a phenylpropanoid-derived polymer found in specific cell types of vascular plants that confers mechanical strength to the cell wall (Boerjan et al., 2003). A previous study has reported that inhibiting lignin biosynthesis could induce the SS in rice (Yoon et al., 2014). However, in our study, the lignin content of the SS-susceptible accession exhibited an increase during the seed developmental stages, and three unigenes related to lignin biosynthesis were upregulated in the SS-susceptible accessions. These results indicated that the increased expression of lignin biosynthesis genes may increase the lignin content in the cell wall, which can strengthen the cell wall but limit its extensibility, thus affecting the degree of SS. To some extent, this result is similar to those of Liu et al. (2022). We can infer from these results that the interaction of these genes could affect the capacity for seed retention during the harvest of stylo, but which gene(s) played the major role in relation to SS still needs to be elucidated.

## Conclusion

To our knowledge, this is the first study regarding the morphological and histological characteristics, hydrolytic enzyme activity, and transcriptome analysis to determine the SS mechanism of stylo accessions. An average SS rate of 76% was found, and the SS phenomenon was common among the stylo accessions. The seed and seed stalk joint is the abscission zone in stylo. Multiplex histology and hydrolytic enzyme activity analysis showed that the tearing of the abscission zone occurs due to the intense enzymatic degradation of PG and CE in the SS-susceptible accession TF0275. In the present study, 525 DEGs that were consistently present in the three groups of DEGs were detected, and many genes involved in plant hormones and cell wall-degrading enzymes were differentially transcribed. A total of 26 genes were associated with the capacity of stylo to shed its seeds, but which gene(s) played the major role in SS still needs further research. Therefore, verifying the functional characterization of these genes in relation to SS might further develop SS-resistant stylo cultivars. This study provides valuable insights for future mechanistic studies of SS in stylo.

## Data availability statement

The data presented in the study are deposited in the https://www.ncbi.nlm.nih.gov/ repository, accession number PRJNA860716.

## Author contributions

GL contributed to the conception and design of the study. XL, JWZ, JXZ, and WS performed the experiments. RH, RD, XD, and PL performed the statistical analysis. XL and JWZ wrote the first draft of the manuscript. All authors contributed to the article and approved the submitted version.

## Funding

This work was supported by the Natural Science Found Program of Hainan (320RC721, 2019RC181); the National Natural Science Foundation of China (no. 31802125); the Specific Research Fund of The Innovation Platform for Academicians of Hainan Province (YSPTZX202020); and the Earmarked Found for CARS (CARS-34).

## Conflict of interest

The authors declare that the research was conducted in the absence of any commercial or financial relationships that could be construed as a potential conflict of interest.

## Publisher’s note

All claims expressed in this article are solely those of the authors and do not necessarily represent those of their affiliated organizations, or those of the publisher, the editors and the reviewers. Any product that may be evaluated in this article, or claim that may be made by its manufacturer, is not guaranteed or endorsed by the publisher.

## Abbreviations

SS, seed shattering; CE, cellulase; PG, polygalacturonase; LL, lobular length; LW, lobular width; PH, plant height; SD, stem diameter; NMB, number of main branch; TKW, 1,000-seed weight; SS, seed shattering; DAF, day after flowering.
