# Supplementary material for: Histological characteristics, cell wall hydrolytic enzyme activity, and transcriptome analysis with seed shattering of Stylosanthes accessions
Source: Front Plant Sci. 2022 Oct 17;13:1018404. doi: 10.3389/fpls.2022.1018404 (PMC9619054; doi:10.3389/fpls.2022.1018404)
Supplement: Supplementary file 1 [file Table_1.docx]

Table S1 **Candidate genes enriched in phenylpropanoid biosynthesis and plant hormone signal transduction pathway**

| KEGG pathway | Gene | Definition | KO id | EC no. | No.All^a^ | No.Up^b^ | No.Down^c^ |
| --- | --- | --- | --- | --- | --- | --- | --- |
| plant hormone signal transduction | | | | | | | |
| Abscisic acid | PP2C | protein phosphatase 2C | K14497 | 3.1.3.16 | 7 | 0 | 7 |
|  | SRK2 | serine/threonine-protein kinase SRK2 | K14498 | 2.7.11.1 | 1 | 0 | 1 |
|  | ABF | ABA responsive element binding factor | K14432 |  | 5 | 1 | 4 |
| Ethylene | ETR, ERS | ethylene receptor | K14509 | 2.7.13 | 5 | 1 | 4 |
|  | EIN2 | ethylene-insensitive protein 2 | K14513 |  | 2 | 1 | 1 |
| Auxin | AUX1, LAX | auxin influx carrier | K13946 |  | 7 | 5 | 2 |
|  | IAA | auxin-responsive protein IAA | K14484 |  | 32 | 24 | 8 |
|  | ARF | auxin response factor | K14486 |  | 2 | 2 | 0 |
|  | GH3 | auxin responsive GH3 gene family | K14487 |  | 1 | 1 | 0 |
|  | SAUR | SAUR family protein | K14488 |  | 32 | 22 | 10 |
| Cytokinine | AHK2_3_4 | arabidopsis histidine kinase 2/3/4 | K14489 | EC:2.7.133 | 12 | 11 | 1 |
|  | AHP | histidine-containing phosphotransfer peotein | K14490 |  | 5 | 3 | 2 |
|  | ARR-A | two-component response regulator ARR-A family | K14492 |  | 1 | 0 | 1 |
| Brassinosteroid | BRI1 | protein brassinosteroid insensitive 1 | K13415 | EC:2.7.10.1 | 7 | 6 | 1 |
|  | BSK | BR-signaling kinase | K14500 | EC:2.7.11.1 | 9 | 4 | 5 |
| Jasmonic acid | JAZ | jasmonate ZIM domain-containing protein | K13464 |  | 7 | 6 | 1 |
| Salicylic acid | NPR1 | regulatory protein NPR1 | K14508 |  | 1 | 1 | 0 |
|  | TGA | transcription factor TGA | K14431 |  | 3 | 0 | 3 |
| phenylpropanoid biosynthesis | PAL | phenylalanine ammonia-lyase | K10775 | 4.3.1.24 | 3 | 1 | 2 |
|  | 4CL | 4-coumarate--CoA ligase | K01904 | 6.2.1.12 | 11 | 7 | 4 |
|  | CCoa-OMT | caffeoyl-CoA O-methyltransferase | K00588 | 2.1.1.104 | 1 | 1 | 0 |
|  | REF1 | coniferyl-aldehyde dehydrogenase | K12355 | 1.2.1.68 | 5 | 3 | 2 |
|  | BGLU | beta-glucosidase | K01188 | 3.2.1.21 | 20 | 15 | 5 |
|  | CCR | cinnamoyl-CoA reductase | K09753 | 1.2.1.44 | 2 | 1 | 1 |
|  | CAD | cinnamyl-alcohol dehydrogenase | K00083 | 1.1.1.195 | 3 | 1 | 2 |
|  | POX | peroxidase | K00430 | 1.11.1.7 | 19 | 16 | 3 |
|  | SOH | shikimate O-hydroxycinnamoyltransferase | K13065 | 2.3.1.133 | 23 | 7 | 16 |

^a^the total number of uni-transcripts analysed

^b^the number of uni-transcripts with expression significantly up-regulated in high seed shattering genotype compared with low seed shattering genotype

^c^the number of uni-transcripts with expression significantly down-regulated in high seed shattering genotype compared with low seed shattering genotype
